# Pathogenic Potential of Coagulase-Positive *Staphylococcus* Strains Isolated from Aviary Capercaillies and Free-Living Birds in Southeastern Poland

**DOI:** 10.3390/ani14020295

**Published:** 2024-01-17

**Authors:** Magdalena Sulikowska, Agnieszka Marek, Łukasz Sebastian Jarosz, Ewelina Pyzik, Dagmara Stępień-Pyśniak, Tomasz Hauschild

**Affiliations:** 1 Eskulap Veterinary Clinic, 37-310 Nowa Sarzyna, Poland; 2 Department of Preventive Veterinary and Avian Diseases, Faculty of Veterinary Medicine, University of Life Sciences in Lublin, 20-950 Lublin, Poland; ewelina.pyzik@up.lublin.pl (E.P.);; 3 Department of Epizootiology and Clinic of Infectious Diseases, Faculty of Veterinary Medicine, University of Life Sciences in Lublin, 20-612 Lublin, Poland; 4 Department of Microbiology and Biotechnology, Faculty of Biology, University of Bialystok, 15-245 Białystok, Poland

**Keywords:** free-living birds, capercaillies, coagulase-positive *Staphylococcus*, virulence genes

## Abstract

**Simple Summary:**

Bacteria of the genus *Staphylococcus* are a component of the natural microbiota of the skin and mucous membranes of animals and humans, but they can also cause endogenous or exogenous infections. They are divided into two groups: coagulase-positive staphylococci (CoPS), which have the ability to clot blood plasma, and coagulase-negative staphylococci (CoNS). The intraspecific diversity of CoPS strains also translates into the variety of features presented by individual strains. A significant problem in veterinary medicine and animal husbandry is the growing resistance of staphylococci to antibiotics. Due to the progress of civilisation, contact between wildlife and the human environment is becoming more frequent, increasing the possibility of the exchange of microbial virulence factors in various ecosystems. The aim of the study was to analyse the occurrence and assessment of the pathogenic potential of individual species of coagulase-positive staphylococci isolated from dead capercaillies kept in aviaries as well as free-living birds in Southeastern Poland. The occurrence of multidrug-resistant CoPS in aviary capercaillies suggests their role in the transmission and spread of resistant strains into the environment. Our results also suggest that free-living birds may be a significant reservoir of enterotoxigenic *Staphylococcus* strains.

**Abstract:**

The aim of the study was to determine the occurrence and characteristics of coagulase-positive *Staphylococcus* strains in the carcasses of wild birds and aviary capercaillies in Southeastern Poland. In total, samples taken from 333 birds were examined. The material consisted of swabs from the internal organs of dead birds (heart, liver, and spleen), the tarsal joints, and mucous membranes (conjunctiva and palatine fissure), as well as from unhatched embryos. The isolated *Staphylococcus* strains were tested for sensitivity to nine antimicrobial agents and the presence of selected virulence genes. An analysis of the similarity of isolates within species was performed using pulsed-field gel electrophoresis (PFGE). The result indicates that coagulase-positive strains accounted for 5.7% and belonged to the species: *Staphylococcus aureus*, *Staphylococcus pseudintermedius,* and *Staphylococcus delphini*. Among isolated strains, 15.8% were multidrug resistant. The most frequently detected virulence genes were *hla* in 58% of isolates and *hlb* and *hld* in 47.4% of isolates. The results of multiplex PCR showed the presence of genes responsible for the production of enterotoxins C, B, E, and J, in single isolates. It can be concluded that coagulase-positive *Staphylococcus* strains accounted for a small percentage of staphylococci isolated from free-living birds in the study area. The occurrence of multidrug-resistant coagulase-positive *Staphylococcus* strains in aviary capercaillies suggests that they play a role in the transmission and spread of resistant strains into the environment. Free-living birds may also be a reservoir of enterotoxigenic *Staphylococcus* strains.

## 1. Introduction

The genus *Staphylococcus* includes several dozen species of immobile Gram-positive cocci that do or do not produce coagulase (CoPS, coagulase-positive staphylococci or CoNS, coagulase-negative staphylococci). Their reservoirs are people and animals, as staphylococci can only multiply in their organisms. These microorganisms are a component of the natural microbiota of the skin and mucous membranes of animals and humans, but they can also cause endogenous or exogenous infections. Coagulase-positive staphylococci are a relatively small but fairly diverse group. Species causing infections in animals include *Staphylococcus aureus*, *Staphylococcus intermedius*, *Staphylococcus delphini*, *Staphylococcus schleiferi* subsp. *coagulans*, *Staphylococcus pseudintermedius*, *Staphylococcus lutrae*, *Staphylococcus agnetis* and coagulase-variable *Staphylococcus hyicus* [1]. The species *S. aureus* is one of the main etiological agents of nosocomial infections and is a very important economic pathogen of animals. Bacteria from the *Staphylococcus intermedius* group (SIG), which include *S. intermedius*, *S. pseudintermedius* and *S. delphini*, colonise the skin and mucous membranes of various animal species, inhabiting the mouth, nose, perineum, and groin (in dogs, horses, and mink) [2]. Two new SIG species, *Staphylococcus cornubiensis* and *Staphylococcus ursi*, have also recently been described and were isolated from human skin in Cornwall, UK, and from healthy black bears (*Ursus americanus*) in Tennessee, USA [3,4]. The remaining species from the SIG group were also isolated from humans with endocarditis, pneumonia, brain abscess, meningitis, and even food poisoning [5,6]. Among CoPS species, those most frequently isolated from birds, especially pigeons (*Columba livia*), are *S. aureus* and species of the SIG group [7]. The numerous virulence factors they produce play an important role in the pathogenesis of infections. The intraspecific diversity of CoPS strains also translates into the variety of features presented by individual strains. Coagulase, involved in the plasma clotting process, is present in all CoPS [8]. Protein A is an important pathogenicity factor and an element of the cell wall of *S. aureus*, but it can also be produced by species of the SIG group [9]. Many CoPS are equipped with numerous enterotoxins and cytolysins (α-, β-, γ-, and δ-haemolysins, and leukotoxins) [10]. Staphylococci have also been shown to produce Panton–Valentine leukocidin (PVL), which is highly species-specific for *S. aureus*. Other leukocidins produced by staphylococci include LukAB/HG, Luk-ED, and LukM. *S. aureus* can also produce a number of bacterial proteins with affinity for host proteins (including fibrinogen, collagen, laminin, transferrin, and lactoferrin), which is of great importance in the initial stage of infection [10]. Like other bacteria, staphylococci may also harbour genes for many other virulence factors. The increasing resistance of staphylococci to antibiotics in recent years is a well-known phenomenon. Studies have shown that as many as 95% of *S. intermedius* strains are resistant to β-lactam antibiotics. An additional threat is an increase in the number of MDR (multidrug-resistant) strains, including strains resistant to fluoroquinolones and mupirocin [11,12,13]. This applies especially to isolates from dogs, poultry, and pigeons [14]. A serious problem is the wide spread of methicillin-resistant strains (MRS). Methicillin-resistant *Staphylococcus aureus* (MRSA) is a significant problem in veterinary medicine and animal husbandry. It is isolated from a wide range of animals, including dogs, cats, rabbits, horses, cattle, pigs, and poultry [15,16]. This phenomenon is associated with the *mec*A gene located within the staphylococcal cassette chromosome *mec* (SCC*mec*). In 2011, in Great Britain, a methicillin-resistant strain of *S. aureus* was isolated from the liver and intestines of a dead free-living finch (*Fringilla coelebs*), in which a new divergent *mec*A gene homologue (mecALGA251) was identified [17]. MRS strains—both coagulase-positive and negative—have also been identified among other species. Methicillin-resistant *Staphylococcus pseudintermedius* (MRSP) strains have been isolated from cats, horses, birds, and humans [11,13,18]. However, the first case of infection with a methicillin-resistant strain of *S. pseudintermedius* in humans was reported in 2006 [18]. Within the species *S. delphini*, there are two phenotypically and genotypically distinct groups of strains (A and B) [19]. These bacteria have been found to be the natural microflora of ferrets, badgers, horses, camels, cows, pigeons, donkeys, and foxes. The pathogenicity of this species has been demonstrated in cows and mink [20,21,22]. There are only a few reports of its isolation from humans [23]. The virulence factors of this species include Panton–Valentine leukocidin, exfoliatin, and enterotoxins [20]. Environmental transmission of CoPS strains of *Staphylococcus* is one of the greatest socioeconomic threats in contemporary medicine.

Due to the lack of data in the available literature on coagulase-positive *Staphylococcus* species in wild birds, especially staphylococci from the SIG group, the aim of the study was to analyse the occurrence of individual species and to assess the pathogenic potential of coagulase-positive *Staphylococcus* strains isolated from dead capercaillies kept in aviaries as well as free-living birds in Southeastern Poland.

## 2. Materials and Methods

### 2.1. Birds

Samples were collected from dead free-living birds from the Podkarpackie province and from capercaillies living in an adaptive aviary in the Wisła Forest District near Żywiec and in the Capercaillie Breeding Centre in the Leżajsk Forest District near Krosno. From November 2019 to August 2023, samples were collected from 117 dead capercaillies (adult birds, chicks, and embryos) and from wild birds belonging to 12 orders and 26 species: Ciconiiformes (white stork, *Ciconia ciconia*, *n* = 57), Galliformes (common pheasant, *Phasianus colchicus*, *n* = 50; black grouse, *Lyrurus tetrix*, *n* = 3), Strigiformes (tawny or brown owl, *Strix aluco*, *n* = 16; long-eared owl, *Asio otus*, *n* = 5; little owl, *Athene noctua*, *n* = 1), Falconiformes (common kestrel, *Falco tinnunculus*, *n* = 13), Anseriformes (mute swan, *Cygnus olor*, *n* = 6; mallard or wild duck, *Anas platyrhynchos*, *n* = 1), Passeriformes (song thrush, *Turdus philomelos*, *n* = 5; blackbird, *Turdus merula*, *n* = 10; meadow pipit, *Anthus pratensis*, *n* = 6; fieldfare, *Turdus pilaris*, *n* = 7; common grosbeak, *Coccothraustes coccothraustes*, *n* = 1; black redstart, *Phoenicurus ochruros*, *n* = 1; Savi’s warbler, *Locustella luscinioides*, *n* = 1; Eurasian blue tit, *Cyanistes caeruleus*, *n* = 1), Piciformes (great spotted woodpecker, *Dendrocopos major*, *n* = 5), Pelecaniformes (grey heron, *Ardea cinerea*, *n* = 3), Apodiformes (common swift, *Apus apus*, *n* = 4), Accipitriformes (common buzzard, *Buteo buteo*, *n* = 10; lesser spotted eagle, *Clanga pomarina*, *n* = 3; white-tailed eagle, *Haliaeetus albicilla*, *n* = 3; Eurasian sparrowhawk, *Accipiter nisus*, *n* = 2), Charadriiformes (white gull, *Larus canus*, *n* = 1), and Bucerotiformes (Eurasian hoopoe, *Upupa epops*, *n* = 1). In total, samples taken from 333 birds were examined. The material for bacteriological tests consisted of swabs taken during autopsy from the internal organs of dead birds (heart, liver, and spleen), the tarsal joints, and mucous membranes (conjunctiva and palatine fissure), as well as from unhatched capercaillie embryos. Swabs were placed in transport media and transported to the laboratory under refrigerated conditions.

### 2.2. Identification of Staphylococcus Strains

The swabs were inoculated onto blood agar medium (Blood LAB-AGAR; Biocorp, Warsaw, Poland) and Chapman’s selective medium (Mannitol Salt LAB-AGAR; Biocorp) and incubated in aerobic conditions at 37 °C for 24–48 h, depending on the bacterial growth rate. Single colonies were transferred to blood agar to isolate pure bacterial cultures, and preliminary bacteriological characterisation of the isolated bacteria was performed, including Gram staining, microscopic examination of cell morphology and motility, and determination of the type of haemolysis. No quantitative colony measurement was performed. Isolated bacteria were stored at −85 °C in 50% (*v*/*v*) glycerol in brain heart broth (BHI; Sigma-Aldrich, St. Louis, MO, USA) until further study.

The species of the isolates were confirmed by matrix-assisted laser desorption/ionisation (MALDI)–time-of-flight mass spectrometry using the IVD MALDI Biotyper (Bruker Daltonik, Bremen, Germany), as described by Marek et al. (2016) [15]. The mass spectra of each isolate were processed with the MALDI Biotyper 3.0 software package (Bruker Daltonics, Hamburg, Germany). The results were shown as the top 10 identification matches along with confidence scores ranging from 0.000 to 3.000, according to the manufacturer’s criteria (www.bruker.com; accessed on: 19 September 2021).

### 2.3. Determination of Bacterial Sensitivity to Antibiotics and Chemotherapeutics Based on Minimum Inhibitory Concentration (MIC)

Selected strains of *Staphylococcus* spp. were analysed for sensitivity to nine antimicrobial agents: benzylpenicillin (0.062–256 μg/mL), amoxicillin (0.125–256 μg/mL), tetracycline (0.125–256 μg/mL), gentamicin (0.250–512 μg/mL), chloramphenicol (0.250–512 μg/mL), erythromycin (0.125–256 μg/mL), enrofloxacin (0.125–256 μg/mL), (Roth, Zielona Góra, Poland), trimethoprim (0.250–128 μg/mL), and sulfamethoxazole (4.75–1216 μg/mL), (Merck KGaA, Darmstadt, Germany). Broth serial microdilution panels of the antibiotics were performed in sterile 96-well spherical-bottom polystyrene titration plates (FL-MEDICAL, Torreglia, Italy). Bacterial suspensions were prepared from morphologically similar colonies grown overnight on nonselective blood agar medium (Blood LAB-AGAR; Biocorp, Warsaw, Poland). An inoculum of 5 × 10^5^ CFU (colony-forming units)/mL suspended in Mueller–Hinton broth (Oxoid Ltd., Basingstoke, UK) was transferred to wells containing serial dilutions of antibiotics (50 μL of bacterial inoculum + 50 μL liquid medium with antibiotic). The plates were incubated at 35 ± 1 °C for 18 ± 2 h. The MIC breakpoint was defined as the lowest concentration of the substance at which no growth of the bacterial strains could be seen. MIC values were interpreted as susceptible, intermediate, or resistant using the current Clinical and Laboratory Standards Institute (CLSI) breakpoint criteria [24]. The specific breakpoints for *S. aureus*, *S. pseudintermedius* and *S. delphini* were as follows: for benzylpenicillin and amoxicillin, susceptibility at ≤0.12 and resistance at ≥0.250 μg/mL; for tetracycline, susceptibility at ≤4 μg/mL, intermediate resistance at 8 μg/mL, and resistance at ≥16 μg/mL; for gentamicin, susceptibility at ≤4 μg/mL, intermediate resistance at 8 μg/mL, and resistance at ≥16 μg/mL; for chloramphenicol, susceptibility at ≤8 μg/mL, intermediate resistance at 16 μg/mL, and resistance at ≥32 μg/mL; for erythromycin, susceptibility at ≤0.5 μg/mL, intermediate resistance at 1–4 μg/mL, and resistance at ≥8 μg/mL; for enrofloxacin, susceptibility at ≤0.5 μg/mL, intermediate resistance at 1–2 μg/mL, and resistance at ≥4 μg/mL; for trimethoprim, susceptibility at ≤2 μg/mL and resistance at ≥4 μg/mL; and for sulfamethoxazole, susceptibility at ≤38 μg/mL and resistance at ≥76 μg/mL. Isolates were tested in duplicate; each result was analysed independently. Quality control was ensured using *S. aureus* ATCC 29213, *Enterococcus faecalis* ATCC 29212, and *S. pneumoniae* ATCC 49619; all quality control results were within published CLSI (2020) MIC limits [24]. Phenotypic identification of the methicillin resistance of *S. aureus* isolates was achieved using the cefoxitin disc diffusion test with a 30 μg disc (Oxoid), according to the Clinical and Laboratory Standards Institute [24].

### 2.4. Molecular Analysis

#### 2.4.1. Genomic DNA Extraction

Single bacterial colonies were inoculated into tryptic soy broth (TSB) medium (CM0129, Oxoid, Ely, UK) and incubated at 37 °C for 12 h. Cells from the overnight culture in TSB medium were harvested by centrifugation at 8000 rpm at 10 °C for 15 min, and the supernatant was then discarded. The pellet was then washed twice with normal saline and centrifuged at 8000 rpm at 10 °C for 15 min. The GeneMatrix Bacterial and Yeast Genomic DNA Purification Kit protocol (EURx, Gdańsk, Poland) was used to isolate bacterial DNA. The bacterial DNA was stained with ethidium bromide, agarose gel electrophoresis was performed, and finally, the fluorescence of the preparations was compared with the fluorescence of the preparation of known concentration.

#### 2.4.2. PCR for Identification of Staphylococcus Isolates and Genotypic Analysis of Virulence

The identity of *S. aureus* isolates was confirmed using a species-specific primer encoding the thermonuclease (*nuc*) gene as one of the key characteristics [25]. Bacterial strains were screened for the presence of virulence-associated genes by PCR amplification with primers at a concentration of 0.04 µmol (Table 1). All isolates were tested for the presence of two resistance genes *mec*A and *mec*C; staphylococcal enterotoxin genes A to L (*sea*, *seb*, *sec*, *sed*, *see*, *seg*, *sei*, *sej*, *seh*, and *sel*); toxic shock syndrome toxin 1 (*tst*); exfoliative toxins A and B (*eta* and *etb*); Panton–Valentine leukocidins (*pvl*); two unrelated leukotoxin components *lukS* and *lukF*; and four haemolysins (*hla*, *hlb*, *hlg,* and *hld*). The presence of the staphylokinase (*sak*) gene was analysed as well. PCR reactions were performed with a ready-to-use reaction solution (Color OptiTaq PCR Master Mix, EURx, Gdańsk, Poland) containing OptiTaq DNA polymerase, optimised reaction buffer, MgCl2, dNTP, two dyes to facilitate tracking of PCR products on the gel, and 100 ng of DNA as template. PCR conditions are detailed in Table 1. For quality control, *S. aureus* RF122, *S. aureus* ATCC43300, *S. aureus* ATCC25923, *S. aureus* ATCC13566, *S. aureus* NCTC13300, FRI913, FRI151m, FRI1169, CCM7056, FRI572, FRI445; *S. haemolyticus* ATCC 29970, *S. epidermidis* ATCC 12228, and *S. epidermidis* ATCC 12228 were used in PCR reactions. PCR products were analysed by gel electrophoresis on a 2% agarose gel and visualised using a transilluminator.

#### 2.4.3. Pulsed—Field Gel Electrophoresis (PFGE)

From each isolate, 250 µL of bacterial suspension from a 16 h culture in brain heart infusion (BHI) broth (Thermo Fisher Scientific, Waltham, MA, USA) was centrifuged for 10 min, 5200× *g*. The cell pellet was suspended in 1 mL of TEN buffer (0.1 M Tris, 0.15 M NaCl, 0.1 M EDTA, pH 8) and centrifuged again (5200× *g*, 10 min). Then the cells were suspended in 0.1 mL of EC lysis buffer (6 mM tris, 1 M NaCl, 100 mM EDTA, 0.5% Brij 58, 0.2 sodium deoxycholate, 0.5 N-lauryl sarcosine sodium, pH 8), and 5 µL of lysostaphin (1 mg/mL) (Sigma, L7386). The cell suspension was mixed with 0.1 mL of 2% LMP agarose (Sigma, A4018). The solution was placed in a cassette to form blocks, left at room temperature for 30 min, and then placed in 1 mL of EC buffer at 37 °C for 3 h. Then the blocks were placed in 1 mL of buffer E (9.5 M EDTA, 1% N-lauryl sarcosine sodium) with 1 mg of protease (Sigma, P6911) and incubated at 37 °C for 24 h. The blocks were washed 5 times in 1 mL of TE buffer (10 mM Tris, 1 mM EDTA, pH 8). Then the DNA blocks were digested in 250 µL of reaction buffer containing 30 U of *Sma*I restriction enzyme (Thermo Fisher Scientific) for 4 h at 30 °C. The blocks were placed in a 1% agarose gel (Sigma, A2929). Electrophoresis was performed in 0.5× TBE buffer for 22 h at 14 °C at 6 V/cm, with a pulsing time ranging from 5 to 40 s. The fragments were separated by electrophoresis in a 1% (*w*/*v*) agarose gel (Sigma-Aldrich, Poznan, Poland) using the CHEF Mapper System (BIO-RAD, Warsaw, Poland). Macrorestriction patterns were examined by cluster analysis using NTSYSpc ver. Software 2.02 (Exeter Software Ltd., New York, NY, USA). The similarity of distances between pulsotypes (PFGE patterns) was calculated using the Dice coefficient, and dendrograms were calculated based on the unweighted pair group method with arithmetic mean (UPGMA). According to the criteria proposed by Tenover et al., isolates whose PFGE pattern differed by more than six restriction fragments (bands) were genetically unrelated and were assigned to different pulsotypes, designated with capital letters. Isolates were considered related if their pulsotype differed by no more than six restriction bands [33].

## 3. Results

### 3.1. Staphylococcus Strains

From samples taken from capercaillies and free-living birds, 334 strains of bacteria belonging to the genus Staphylococcus were identified. The Staphylococcus strains belonged to 24 species. Coagulase-positive strains accounted for 5.7%, while the remaining 94.3% were coagulase-negative species. The percentages of strains belonging to individual species were as follows: *S. sciuri* 45.2%, *S. lentus* 8%, *S. xylosus* 7.8%, *S. equorum* 6.6%, *S. aureus* 3.9%, *S. kloosii* 3.3%, *S. saprophyticus* 3%, *S. chromogenes* 2.7%, *S. vitulinus* 2.7% *S. epidermidis* 2.7%, *S. succinus* 2.4%, *S. cohnii* 2.1%, *S. haemolyticus* 2.1%, *S. gallinarum* 1.8%, *S. pseudintermedius* 1.5%, *S. warneri* 1.2%, *S. simulans* 0.6%, *S. felis* 0.6%, *S. delphini* 0.3%, *S. condimenti* 0.3%, *S. nepalensis* 0.3%, *S. arlettae* 0.3%, *S. pasteuri* 0.3%, and *S. stepanovicii* 0.3%. Coagulase-positive Staphylococcus strains belonging to the species *S. aureus*, *S. pseudintermedius,* and *S. delphini* were further examined. Log(score) values for these isolates obtained in MALDI-TOF mass spectrometry were higher than 2000, which indicates a high probability of correct identification to the species level (Table 2).

### 3.2. Susceptibility of Isolated Strains to Selected Antimicrobial Agents

Analysis of the antimicrobial resistance data presented in Table 2 showed that nine of nineteen coagulase-positive staphylococcal strains were fully susceptible to the antimicrobials tested. Three strains (SP3, SA9, and SA11) were resistant to three or more groups of antibiotics, while two strains (SP1 and SP4) were resistant to two groups of antibiotics. The highest number of resistant strains was observed for beta-lactam antibiotics (*n* = 7). Table 2 shows the lowest concentration of the antibacterial agent that completely inhibited visible growth. None of the results of the tests of quality control strains were outside the ranges defined by CLSI [24].

#### Resistance of *S. aureus* Strains to Oxacillin

A surrogate test for the resistance of *S. aureus* strains to oxacillin using the cefoxitin (30 µg) disc diffusion method showed that the growth inhibition zone for all 13 strains was equal to or greater than 22 mm, which means that all strains tested were susceptible.

### 3.3. Molecular Analysis

#### 3.3.1. Molecular Identification of Staphylococcus Isolates and Genotypic Analysis of Virulence

The presence of the thermonuclease (*nuc*) gene was confirmed in all 13 *S. aureus* isolates. The presence of the *mec*A and *mec*C genes was not detected in any of the 19 coagulase-positive *Staphylococcus* strains tested. Among the virulence genes analysed, the α-toxin encoding gene (*hla*) was found in 11/19 (58%) of the isolates and was the most frequently detected virulence gene. The presence of the *hlb* and *hld* genes was demonstrated in 9/19 isolates. Only one *S. aureus* strain had the *hlg* gene. The results of multiplex PCR for ten enterotoxins (A–L) showed that the genome of one of the *S. pseudintermedius* strains contained the gene responsible for the production of enterotoxin C, and one *S. aureus* strain had the gene responsible for the production of enterotoxin B. Two *S. aureus* strains had the gene responsible for the production of enterotoxin G, and one *S. aureus* strain showed the presence of the two genes for enterotoxins E and J. The presence of the *see* gene was demonstrated in the *S. delphini* isolate. None of the *Staphylococcus* strains had the genes responsible for the production of enterotoxins A, D, H, I, or L (Table 2). However, the presence of the *sak* gene was confirmed in one strain of *S. aureus*.

#### 3.3.2. Pulsed Field Gel Electrophoresis (PFGE)

**Figure 1:**
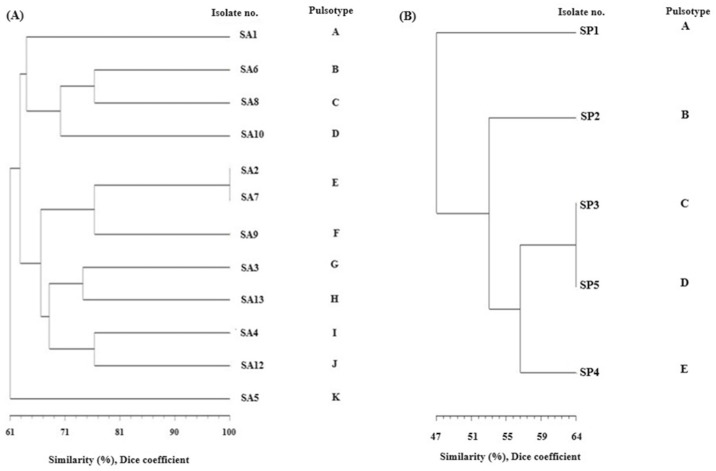
Dendrogram showing the level of similarity of *Sma*I restriction PFGE patterns of the genomic DNA of *S. aureus* (**A**) and *S. pseudintermedius* (**B**) isolates.

**Figure 2:**
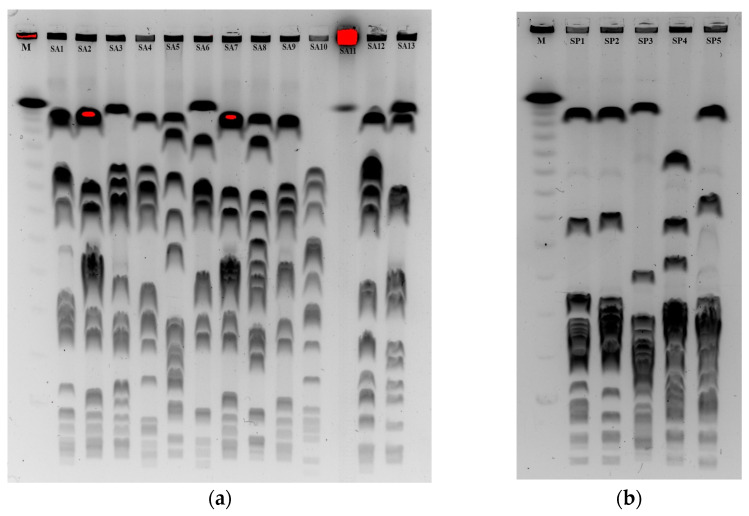
PFGE patterns of *S. aureus* (**a**) and *S. pseudointermedius* (**b**) isolates digested with *Sma*I restriction enzyme. Strains belonging to the same macrorestriction type and the strain that was not digested with the *Sma*I enzyme are marked in red.

Digestion of genomic DNA with *Sma*I endonuclease revealed the presence of 11 different PFGE patterns (pulsotypes A–K) among 12 *S. aureus* strains since 1 strain (SA11) was not typable. Two strains (SA2 and SA7) showed the same pulsotype E and were undistinguishable by their PFGE patterns. The remaining 10 pulsotypes were represented by a single strain only (Figure 1A and differed by more than seven bands, which means that they were not related according to Tenover’s criteria (Figure 2a)) [33]. Analysis of the phylogenetic relatedness of *S. pseudintermedius* strains revealed five pulsotypes (A–E), indicating that all strains differed significantly and were not related according to Tenover’s criteria (Figure 1B and Figure 2b).

## 4. Discussion

Among the isolated *Staphylococcus* strains, only three coagulase-positive species were identified in our study, constituting 5.7% of all isolates obtained from dead capercaillies and free-living birds. The most numerous species was *S. aureus*. Reports on the occurrence of coagulase-positive staphylococci in wild birds from the last 10 years were analysed. Ruiz-Ripa et al. (2019) identified 26 (8.3%) coagulase-positive staphylococci among 324 tracheal samples collected from healthy wild birds with occasional or obligatory scavenging habits in Spain (a cinereous vulture, a red kite, and magpies). Among the isolated strains, the authors identified two CoPS species: *S. aureus* (*n* = 15 isolates) and *S. delphini* (*n* = 12 isolates) [21]. In another study, analysing samples taken from the trachea of 92 storks located in Spain, *S. aureus* was isolated in 23.8% of the birds. Importantly, the occurrence of *S. aureus* was higher in birds from landfills (55.8%) than in those from natural habitats (16.3%) [34]. Examination of samples collected from birds of prey in Portugal showed that 37.5% of them were carriers of staphylococci, of which six species were identified. Only one coagulase-positive species (*S. aureus*) was isolated from the common buzzard (*Buteo buteo*). The remaining strains belonged to coagulase-negative species [35]. Little is known about the prevalence of SIG species in free-living birds, particularly *S. pseudintermedius*, especially given that it is considered to be the dominant staphylococcal etiological agent of pyoderma, ear infections, and urinary tract infections in dogs [36,37]. The new coagulase-positive species *S. pseudintermedius* was introduced relatively recently (in 2005), based on molecular analyses of zoonotic strains phenotypically and genotypically similar to *S. intermedius* [37]. The literature data indicate that it occurs mainly in *Canidae* as an element of the physiological microbiota of the skin and mucous membranes, but it has also been isolated from cats, parrots, and humans [36,37]. Most of the SIG group of staphylococci isolated from birds, especially from pigeons, have been shown to belong to the species *S. intermedius* and *S. delphini* [38]. It is very difficult to differentiate between *S. intermedius*, *S. delphini*, and *S. pseudintermedius* phenotypically. For a long time, *S. pseudintermedius* was often incorrectly identified as *S. aureus* or *S. intermedius* in many laboratories, especially since biochemical features specific to the species *S. pseudintermedius* have not been determined. In our study, six strains of *S. aureus* were isolated from capercaillies in an adaptive aviary, which constituted 5.1% of all examined capercaillies, and from seven free-living birds, which constituted 3.2% of all those examined. *S. pseudintermedius* was isolated from 2.6% of capercaillies and less than 1% of free-living birds, while *S. delphini* was isolated from one capercaillie. 

Reliable identification of coagulase-positive staphylococci isolated from animals and humans has become particularly important since the occurrence of highly pathogenic strains has been confirmed in this group of microorganisms. Moreover, MIC breakpoints for some antibiotics, e.g., oxacillin, used to determine the resistance of staphylococci to methicillin, vary depending on the *Staphylococcus* species [24]. Rapid bacterial identification systems based on mass spectrometry, such as MALDI TOF (matrix-assisted laser desorption/ionisation time-of-flight), may be useful for identifying species belonging to the SIG group if the databases used include these bacteria. The sensitivity of the MALDI TOF method for identifying *S. pseudintermedius* has been estimated at 78%, and its specificity at 97% [7]. However, in recently published research by Sawhney et al. (2023), a comparative analysis of the effectiveness of whole genome sequencing (WGS) and MALDI-TOF MS in identifying staphylococci from the SIG group among 493 strains of *S. pseudintermedius* and 7 strains of *S. delphini* showed that MALDI-TOF provides 99.8% and 100% precision in identifying an isolate as *S. pseudintermedius* and *S. delphini*, respectively [39]. In our study, log(score) values for *S. pseudintermedius* and *S. delphini* isolates obtained in MALDI-TOF mass spectrometry were higher than 2.0, which indicates a high probability of correct identification to the species level (Table 2). 

Restriction analysis of chromosomal DNA in combination with pulse electrophoresis (PFGE) is the most frequently used method for studying the epidemiology of locally occurring *Staphylococcus* strains. The most commonly used restriction enzymes are *Sma*I and *Csp*I. Strains that have the same restriction patterns are classified as the same macrorestriction type. Strains with differences in one or two bands corresponding to changes in the genome (insertions, deletions, or point mutations) are assigned to subtypes, and isolates differing in three or more bands are assigned to different PFGE types [33]. In this way, it is possible to establish genetic relationships between isolates in epidemiological studies [40]. In our study, 12 of the *S. aureus* isolates were typeable by *Sma*I enzyme digestion. One *S. aureus* (SA11) strain was not digested by this enzyme. It is possible that the activity of *Sma*I is blocked due to methylation of the restriction site [41]. Interestingly, analysis of the genetic material of 13 *S. aureus* strains using a rare-cutting restriction enzyme and separation of the resulting DNA fragments revealed that two isolates (SA2 and SA7) had the same *Sma*I-PFGE patterns and belonged to the same macrorestriction type. Isolates were obtained from birds of prey from the same area: from a wound in a common kestrel (*Falco tinnunculus*) and from the mucous membrane of the palatal fissure of a buzzard (*Buteo buteo*). It was also determined that the macrorestriction patterns of the remaining *S. aureus* and *S. pseudintermedius* isolates differed by more than seven bands, which means that they were not related according to Tenover’s criteria (Figure 1 and Figure 2a,b) [33].

*Staphylococcus aureus* is a species with significant pathogenic potential, often isolated from clinical material from humans and animals. However, features previously considered typical of this species may also be present in other coagulase-positive species, which may be of importance during infections. The presence of *S. aureus* strains in the tissues and organs of wild birds is quite common [17,34,35,42,43,44,45,46,47,48,49,50]. Additionally, *S. aureus* produces several virulence factors that enable the bacteria to survive unfavourable conditions in the host and to damage biological membranes, causing cell death. *S. aureus* bacteria precisely control the expression of virulence factors, including haemolysins, leukocidins, proteases, exfoliative toxins, enterotoxins, and immunomodulatory factors [28,29]. 

In a study by Silva et al. (2022) of throat and cloacal swabs collected from 114 individuals belonging to various species of nocturnal birds of prey (*Strix aluco*, *Tyto alba*, *Athene noctua,* and *Bubo bubo*) in Central and Northern Portugal, 25 strains of CoPS were isolated. Most of them (twenty-three strains) were identified by MALDI-TOF as *S. aureus*, while two strains belonged to the species *S. pseudintermedius* [50]. In our study, five strains of *S. pseudintermedius* were isolated from samples collected from the conjunctiva, joints, and liver of three capercaillies, a meadow pipit and a white stork. One *S. delphini* isolate was obtained from the yolk sac of a dead capercaillie chick (Table 2). *S. delphini* has been isolated from many animal species in which it caused infections [21,22]. This species can be divided into two genetically distinct clusters, *S. delphini* group A and group B, for which animal host specificity may exist. Some research suggests that the family *Mustelidae* may be the natural host of *S. delphini* group A, while *S. delphini* group B is more frequently detected in wild and domestic pigeons, although this is still under investigation [36,38]. In our study, the group membership of the isolated *S. delphini* strain was not determined. The *S. delphini* strain was fully sensitive to nine of the antimicrobials tested. PCR testing revealed the presence of two haemolysin genes, *hlb* and *hld*, in the genome of this isolate, as well as the see gene responsible for the production of enterotoxin E.

*S. pseudintermedius* virulence factors, like those produced by *S. aureus*, promote colonisation and infection [19]. Reports indicate that *S. pseudintermedius* is an opportunistic pathogen that can cause infections of the skin (purulent dermatitis, otitis externa, wound infections, or abscesses), and other tissues and body cavities [19,39,51,52]. The ability to haemolyse blood cells by producing cytolysins is a feature observed in various species of staphylococci. These are important factors in the pathogenicity of these bacteria [10]. The pathogenic potential of *S. pseudintermedius* is determined by virulence factors including coagulase, protein A, protease, enterotoxins, TSST-1 (toxic shock syndrome toxin), exfoliative toxin SIET, and cytolysins, i.e., leukotoxin Luk-I and haemolysins [19,39]. PCR analysis for virulence determinants of strains isolated from capercaillies and free-living birds showed that genes involved in avoidance and invasion (*hla* and *hlb*) were the most common in *S. aureus* isolates. The α-toxin encoding gene (*hla*) was found in 10/13 (77%) of the *S. aureus* isolates, and it was the most frequently detected virulence gene. The presence of the *hlb* and *hld* genes was found in 9/13 and 7/13 *S. aureus* strains, respectively. In the case of other *Staphylococcus* strains, the presence of *hla* and *hld* genes was confirmed in one strain of *S. pseudintermedius* isolated from the joint of a capercaillie, and the presence of *hlb* and *hld* genes in an isolate of *S. delphini* from the yolk sac of a capercaillie. Silva et al. (2022), analysing strains of *S. aureus* and *S. pseudintermedius* isolated from nocturnal birds of prey in Portugal, also detected genes *hla* and *hlb* encoding virulence factors. All isolates carried at least one virulence gene, with the *hla* gene present in all isolates and the *hlb* gene in fifteen isolates. Four *S. aureus* isolates contained the *sak* gene [50]. As some authors point out, in the case of *S. pseudintermedius* strains, β-haemolysin is produced constitutively, while δ- and α-toxin are rarely produced [20,51]. The results of research by Afzal et al., 2022 on staphylococci isolated from humans indicate that the *hlg* gene is more common in infectious strains of *S. aureus* than in non-infectious strains [53]. Alpha-haemolysin is the most commonly studied of the *S. aureus* cytotoxins because it is produced by numerous strains and can damage a wide range of mammalian cells. In our study, the presence of the *hlg* gene was detected in only one of the *S. aureus* strains, which was isolated from the palatal fissure of a capercaillie. 

Genes located on mobile genetic elements, such as pathogenicity islands or lysogenic bacteriophages, may be carriers of virulence factors, including TSST, some enterotoxins, or Panton–Valentine leukocidin (PVL), while genes of other virulence factors such as staphylokinase (SAK) are integrated into the bacterial chromosome [54,55]. The most important virulence factors for diagnostics include enzymes staphylokinase (plasminogen activator) and haemolysins, primarily β-toxin, also called sphingomyelinase, which destroys the cell membrane of macrophages, leukocytes and erythrocytes [31,53]. Staphylokinase, encoded by the *sak* gene, can be produced by lysogenic strains of both *S. aureus* and non-aureus staphylococci [54]. PCR testing for the staphylokinase (*sak*) gene in CoPS strains from capercaillies and free-living birds showed its presence in one strain of *S. aureus* isolated from the song thrush in the palatal fissure. The role of this protein as a virulence factor of staphylococci is assumed to stem from its interaction with plasminogen and defensins. The binding of staphylokinase to plasminogen may affect bacterial invasion into the host tissues. The activity of virulence factors encoded by bacteriophages, such as staphylokinase, is assumed to be specific to human cells, which indicates a close relationship between the host and the pathogen [56]. Some authors suggest that *Staphylococcus* strains of animal origin carry *sak*-containing phages less frequently than human isolates [57]. 

PVL is a two-component, β-barrel pore-forming toxin [58]. PVL and γ-haemolysin are toxins that act on cell membranes through the synergy of two pore-forming proteins. A small percentage of *S. aureus* isolates produce PVL [55]. PVL is also the most leukocytolytic toxin and causes skin necrosis [59]. To date, PVL-positive *S. aureus* strains have been documented in companion animals but have rarely been found in other animal species [16]. The presence of genes responsible for the production of toxic shock syndrome toxin-1 (TSST-1), Panton–Valentine leukocidin (PVL), or exfoliative toxins was not confirmed in any CoPS strains isolated from capercaillies and free-living birds. 

Staphylococcal enterotoxins (SE), classified as superantigens, are exotoxins that are synthesised by many *Staphylococcus* species during the log phase of growth or during the transition from the exponential phase to the stationary phase. Thus far, 33 enterotoxins (SEs) and SE-like toxins (SEls) have been described. The most frequently identified toxins (>75%) worldwide are SEA to SEE enterotoxins, referred to as “classic SE” [60]. They are mainly produced by coagulase-positive staphylococci, but the genes responsible for the production of enterotoxins have also been identified in coagulase-negative strains isolated from birds (poultry) [61]. Genes encoding enterotoxins (*se*) are most often located on mobile genetic elements, such as plasmids, prophages, or pathogenicity islands [28]. Screening of CoPS strains isolated from capercaillies and free-living birds for 10 enterotoxin genes (classic A–E and new G–L) confirmed the presence of enterotoxin genes in 4 of 13 *S. aureus* isolates. However, PFGE analysis showed that two *S. aureus* strains (SA2 and SA7) carrying the *seg* gene were closely related but isolated from two different bird species. Moreover, one strain of *S. aureus*, isolated from the spleen of a common buzzard, had the gene responsible for the production of enterotoxin B, and the presence of two genes, *se*e and *sej*, was confirmed in one strain isolated from a song thrush. In the genome of one strain of *S. pseudintermedius*, isolated from a capercaillie, the gene responsible for the production of classical enterotoxin C was found. The *S. delphini* strain isolated from a capercaillie also had the gene for a classical enterotoxin (*see*). Staphylococcal strains isolated from wild animals are rarely characterised in terms of potential enterotoxicity. Moreover, unlike *S. aureus*, no enterotoxin screening has been performed at the genome level for SIG species. The few studies of veterinary isolates of *S. intermedius* from horses, dogs, and cats and of human strains showed that just over 10% of the strains were positive for the enterotoxin genes [60].

An additional threat is the increase in the number of drug-resistant strains recorded in recent years, including methicillin-resistant and multi-drug-resistant (MDR) strains of *Staphylococcus* [52], especially those resistant to fluoroquinolones, isolated from companion and farm animals [62]. Resistance genes are most often located on mobile genetic elements (MGEs), such as the staphylococcal cassette chromosome (SCC*mec*), plasmids, and transposons [58]. Methicillin resistance in various staphylococcal species isolated from animals has proven to be a serious therapeutic challenge over the last decade. *S. pseudintermedius* strains carrying the *mec*A gene are referred to as MRSP (methicillin-resistant *S. pseudintermedius*) and, like MRSA (methicillin-resistant *Staphylococcus aureus*), are usually multi-drug resistant, because SCC*mec* often contains resistance genes to other antibiotics and chemotherapeutics as well. Cases of MRSP infections have been reported, especially in dogs, cats, birds, horses, and humans [11]. However, studies of the role of wildlife as a reservoir of MRSA and other methicillin-resistant CoPS are limited, in comparison with humans and domestic animals. In a study by Silva et al. (2022), on strains isolated from nocturnal birds of prey, only one of twenty-three *S. aureus* isolates was resistant to cefoxitin and contained the *mec*C gene. Two isolates showed phenotypic resistance to macrolides and lincosamides. However, two *S. pseudintermedius* isolates were sensitive to all antibiotics tested, but the presence of the *mec*A gene was identified in one of them [50]. In our study, the analysis of the presence of the *mec*A gene and a novel *mec*A homologue, *mec*C, did not show the presence of these genes in any of the 19 isolates from capercaillies and free-living birds in Southeastern Poland. The study of phenotypic resistance to antimicrobials using the broth microdilution method showed that 36.8% of the coagulase-positive strains were resistant to beta-lactam antibiotics (penicillin and amoxicillin), 26.3% were resistant to chloramphenicol, and 15.8% to tetracycline, erythromycin, and trimethoprim. However, three of the nineteen strains were multidrug-resistant (MDR). MDR strains were isolated from capercaillies kept in adaptive aviaries. This phenomenon should probably be linked to the fact that capercaillies kept in aviaries have closer contact with the human environment. None of the CoPS strains showed phenotypic resistance to gentamicin, enrofloxacin, or sulfamethoxazole. The pathogenic potential of the bacterial strains isolated from wild birds obviously varies depending on their environment (urban or farmland), which is undoubtedly linked to the presence of people, farm animals, hospitals, and production plants. Additionally, migratory birds such as the meadow pipit and white stork may play a key role in the spread of virulence genes across habitats and regions, especially as they use highly anthropogenic habitats such as landfills and urban areas, but empirical data remain scarce. In the latest study of samples from the breeding sites of wild birds living in parks and near hospital waste dumps in Islamabad (Pakistan), 174 isolates were biochemically identified as *S. aureus*. Antimicrobial sensitivity testing showed that nearly 29% of them were multidrug-resistant, and half of them were designated as MRSA. The majority of MDR isolates came from faecal samples, collected from the vicinity of hospitals [63]. The available literature contains reports of the occurrence of MRSA strains isolated from bird species such as white stork, cinereous vulture, Eurasian griffon vulture, and magpie in Spain; lesser yellowlegs in the United States; and common chaffinch in Scotland, and the *mec*A homologue (*mec*A LGA251) has been found in strains isolated from Canada goose faecal samples [17,21,34,45,47,49]. According to studies on *S. aureus*, the population structure may vary regionally [15,34,43]. Analysis of coagulase-positive strains from wild birds in Spain showed that among the collection of 15 *S. aureus* isolates, 13 were MRSA. Moreover, the MRSA isolate was *mec*A-positive, and 12 MRSA isolates harboured the *mec*C gene [21].

Due to the progress of civilisation, contact between wildlife and the human environment is becoming more frequent, increasing the possibility of the exchange of microbial virulence factors in various ecosystems. There are many reports of the pathogenic potential of staphylococci isolated from humans, companion animals, and farm animals. However, populations of coagulase-positive staphylococci found in wild animals, especially birds, in various regions of the world are still poorly characterised.

## 5. Conclusions

Coagulase-positive *Staphylococcus* strains constituted only a small percentage (5.7%) of staphylococcal species isolated from dead capercaillies and from various species of free-living birds in Southeastern Poland. 

Although staphylococci from the SIG group are rarely isolated from free-living birds, our results indicate that *S. pseudintermedius* and *S. delphini* may be present on the mucous membranes and internal organs of capercaillies and other species of wild birds.

The occurrence of multidrug-resistant CoPS in aviary capercaillies suggests their role in the transmission and spread of resistant strains into the environment. The largest number of resistant strains isolated from capercaillies and free-living birds was found for beta-lactam antibiotics. The study results clearly indicate the need for monitoring resistant bacteria in wild birds and other wildlife.

The analysis of virulence determinants of strains isolated from capercaillies and free-living birds showed that genes involved in avoidance and invasion (*hla* and *hlb*) were the most common in the isolates. Enterotoxin production by CoPS isolates from free-living birds has not previously been assessed. We identified *S. aureus* and SIG group isolates carrying genes for both classical and novel enterotoxins. Our results suggest that free-living birds may be a significant reservoir of enterotoxigenic *Staphylococcus* strains.

## Figures and Tables

**Table 1:** Nucleotide sequences and sizes of PCR products of amplified genes.

Primer Name	Oligonucleotide Sequence (5′-3′) *	Amplicon Size (bp)	Target Gene	PCR Conditions	Reference
*nuc*-F *nuc*-R	GCGATTGATGGTGATACGGTT AGCCAAGCCTTGACGAACTAAAGC	270	*nuc*	94 °C, 5 min, 37 cycles of 94 °C for 1 min, 55 °C for 30 s, 72 °C for 1 min, final extension 72 °C for 7 min	[25]
*mec*A-F *mec*A-R	AAAATCGATGGTAAAGGTTGGC AGTTCTGGCACTACCGGATTTGC	533	*mec*A	94 °C, 5 min, 40 cycles of 94 °C for 1 min, 58 °C for 1 min, 72 °C for 2 min, final extension 72 °C for 5 min	[26]
*mec*C-F *mec*C-R	GAA AAA AAG GCT TAG AAC GCC TC GAA GAT CTT TTC CGT TTT CAG C	138	*mec*C	94 °C, 15 min, 30 cycles of 94 °C for 30 s, 59 °C for 1 min, 72 °C for 1 min, final extension for 10 min	[27]
*sea*-F *sea*-R	ACGATCAATTTTTACAGC TGCATGTTTTCAGAGTTAATC	544	Enterotoxins	94 °C, 5 min, 35 cycles of 94 °C for 2 min, 57 °C for 2 min, 72 °C for 1 min, final extension 72 °C for 7 min	[28]
*seb*-F *seb*-R	GAATGATATTAATTCGCATC TCTTTGTCGTAAGATAAACTTC	416			
*sec*-F *sec*-R	GACATAAAAGCTAGGAATTT AAATCGGATTAACATTATCCA	257			
*sed*-F *sed*-R	TTACTAGTTTGGTAATATCTCCTT CCACCATAACAATTAATGC	334			
*see*-F *see*-R	ATAGATAAAGTTAAAACAAGCAA TAACTTACCGTGGACCC	170			
*seg*-F *seg*-R	CCACCTGTTGAAGGAAGAGG TGCAGAACCATCAAACTCGT	432		94 °C for 2 min, 30 cycles of 94 °C for 25 s, 50 °C for 20 s, 72 °C for 40 s, final extension 72 °C for 6 min	[29]
*seh*-F *seh*-R	TCACATCATATGCGAAAGCAG TCGGACAATATTTTTCTGATCTTT	463			
*sei*-F *sei*-R	CTCAAGGTGATATTGGTGTAGG CAGGCAGTCCATCTCCTGTA	529			
*sej*-F *sej*-R	GGTTTTCAATGTTCTGGTGGT AACCAACGGTTCTTTTGAGG	306			
*sel*-F *sel*-R	CACCAGAATCACACCGCTTA CTGTTTGATGCTTGCCATTG	240			
*tst*-F *tst*-R	ACCCCTGTTCCCTTATCATC TTTTCAGTATTTGTAACGCC	326	*tsst-1*	95 °C, 5 min, 30 cycles of 94 °C for 1 min, 55 °C for 30 s, 72 °C for 1 min, final extension at 72 °C for 5 min	[12]
*pvl*-F *pvl*-R	ATCATTAGGTAAAATGTCTGGACATGATCCA GCATCAASTGTATTGGATAGCAAAAGC	433	*pvl*		
*eta*-F *eta*-R	GCAGGTGTTGATTTAGCATT AGATGTCCCTATTTTTGCTG	93	*eta* *etb*		
*etb*-F *etb*-R	ACAAGCAAAAGAATACAGCG GTTTTTGGCTGCTTCTCTTG	226			
*lukS*-F *lukS*-R	TGTAAGCAGCAGAAAATGGGG GCCCGATAGGACTTCTTACAA	503	*lukS*	94 °C, 3 min, 35 cycles of 94 °C for 1 min, 57 °C for1 min, 72 °C for 1 min, final extension 72 °C for 7 min	[30]
*lukF*-F *lukF*-R	CCTGTCTATGCCGCTAATCAA AGGTCATGGAAGCTATCTCGA	572	*lukF*		
*sak*-F *sak*-R	TGAGGTAAGTGCATCAAGTTCA CCTTTGTAATTAAGTTGAATCCAGG	403	*sak*	94 °C, 15 min, 35 cycles of 94 °C for 30 s, 55 °C for 30 s, 72 °C for 2 min	[31]
*hla*-F *hla*-R	CTGATTACTATCCAAGAAATTCGATTG CTTTCCAGCCTACTTTTTTATCAGT	209	Haemolysins	95 °C for 5 min, 30 cycles of 95 °C for 1 min, 58 °C for 1 min and 72 °C for 2 min, final extension of 72 °C for 10 min	[32]
*hlb*-F *hlb*-R	GTGCACTTACTGACAATAGTGC GTTGATGAGTAGCTACCTTCAG	309			
*hld*-F *hld*-R	AAGAATTTTTATCTTAATTAAGGAAGGAGTG TTAGTGAATTTGTTCACTGTGTCGA	111			
*hlg*-F *hlg*-R	GTCAYAGAGTCCATAATGCATTTAA CACCAAATGTATAGCCTAAAGTG	535			

* F—forward primer; R—reverse primer. The sets of primers were synthesised by Genomed S.A, Warsaw, Poland. The concentration of primers was 0.04 µmol.

**Table 2:** Susceptibility to antibiotics and chemotherapeutics (MIC) and the presence of virulence genes in CoPS strains.

*Staphylococcus* Species		MALDI-TOF MS Biotyper Log (Score)	Bird Species	Organ	MIC Breakpoints μg/mL									Presence of Virulence Genes
					Penicillin	Amoxicillin	Gentamicin	Chloramphenicol	Tetracycline	Enrofloxacin	Erythromycin	Trimethoprim	Sulfamethoxazole	
*S. pseudintermedius*	SP1	2.100	Capercaillie	Conjunctiva	≤0.062	≤0.062	≤0.5	≥32	≤0.125	≤0.125	≤0.125	≥16	≤19	-
	SP2	2.096	Capercaillie	Joint	≤0.062	≤0.062	≤0.5	≤4	≤0.125	≤0.125	≤0.5	≤0.5	≤38	*sec*, *hla*, *hld*
	SP3	2.367	Capercaillie	Liver	≥64	≥1	≤0.250	≥64	≥32	≤0.5	≥256	≤1	≤38	-
	SP4	2.168	Meadow pipit	Joint	≤0.125	≤0.125	≤0.250	≥32	≤0.250	≤0.125	≥16	≤0.5	≤38	-
	SP5	2.008	White stork	Conjunctiva	≥16	≥0.250	≤0.250	≤4	≤0.125	≤0.5	≤0.250	≤1	≤19	-
*S. aureus*	SA1	2.205	Capercaillie	Embryo	≥32	≥64	≤1	≤4	≤0.125	≤0.125	≤0.5	≤0.5	≤19	*nuc*, *hla*, *hld*
	SA2	2.260	Common kestrel	Wound	≤0.062	≤0.062	≤1	≤4	≤0.125	≤1	≤0.5	≤2	≤38	*nuc*, *seg*, *hla*, *hlb*
	SA3	2.249	Song thrush	Palatal fissure	≥8	≥4	≤1	≤8	≤0.250	≤0.250	≤0.5	≤0.250	≤19	*nuc*, *see*, *sej*, *sak*
	SA4	2.314	Capercaillie	Conjunctiva	≤0.062	≤0.062	≤1	≤4	≤0.250	≤0.125	≤0.5	≤0.5	≤38	*nuc*, *hla*, *hlb*, *hld*
	SA5	2.419	Capercaillie	Embryo	≤0.125	≤0.125	≤0.5	≤4	≤0.125	≤0.125	≤0.250	≤2	≤19	*nuc*, *hla*, *hlb*
	SA6	2.363	Common kestrel	Palatal fissure	≤0.062	≤0.062	≤0.5	≤8	≤0.250	≤0.125	≤0.5	≤1	≤19	*nuc*, *hla*, *hlb*, *hld*
	SA7	2.054	Common buzzard	Palatal fissure	≤0.062	≤0.062	≤1	≤4	≤0.125	≤0.5	≤0.5	≤2	≤9,5	*nuc*, *seg*, *hla*, *hlb*
	SA8	2.146	Capercaillie	Liver	≤0.062	≤0.062	≤0.250	≤4	≤0.5	≤0.125	≤0.5	≤0.5	≤19	*nuc*, *hla*, *hlb*, *hld*
	SA9	2.326	Capercaillie	Embryo	≤0.062	≤0.062	≤1	16	≥16	≤0.250	≤0.5	≥16	≤38	*nuc*, *hla*, *hlb*
	SA10	2.359	Common kestrel	Liver	≥0.5	≥0.5	≤1	≤4	≤0.5	≤0.125	≤0.5	≤0.5	≤19	*nuc*, *hla*, *hlb*, *hld*
	SA11	2.230	Capercaillie	Palatal fissure	≥4	≥2	≤1	≥256	≥256	≤0.125	≥256	≥8	≤38	*nuc*, *hlg*
	SA12	2.030	Common buzzard	Spleen	≤0.125	≤0.125	≤1	≤8	≤1	≤0.125	≤0.5	≤2	≤38	*nuc*, *seb*, *hld*
	SA13	2.342	Common buzzard	Joint	≥0.250	≥0.250	≤1	≤8	≤0.250	≤0.125	≤0.5	≤0.5	≤19	*nuc*, *hla*, *hld*
*S. delphini*	SD1	2.028	Capercaillie	Yolk sac	≤0.062	≤0.062	≤0.250	≤4	≤0.125	≤0.125	≤0.5	≤2	≤38	*see*, *hlb*, *hld*

## Data Availability

The data presented in this study are available in article.
